# HHLA2 in intrahepatic cholangiocarcinoma: an immune checkpoint with prognostic significance and wider expression compared with PD-L1

**DOI:** 10.1186/s40425-019-0554-8

**Published:** 2019-03-18

**Authors:** Chu-Yu Jing, Yi-Peng Fu, Yong Yi, Mei-Xia Zhang, Su-Su Zheng, Jin-Long Huang, Wei Gan, Xin Xu, Jia-Jia Lin, Juan Zhang, Shuang-Jian Qiu, Bo-Heng Zhang

**Affiliations:** 1 Fudan University Shanghai Cancer Center, 270 Dong-An Road, Shanghai, 200032 People’s Republic of China; 2 The Liver Cancer Institute, Zhongshan Hospital and Shanghai Medical School, Fudan University, Key Laboratory for Carcinogenesis and Cancer Invasion, The Chinese Ministry of Education, 180 Fenglin Road, Shanghai, 200032 People’s Republic of China; 3 Center for evidence-based medicine, Shanghai Medical School, Fudan University, Shanghai, 200032 People’s Republic of China; 4 Department of breast surgery, The Obstetrics & Gynecology Hospital of Fudan University, 419 Fangxie Road, Shanghai, 200090 People’s Republic of China

**Keywords:** Intrahepatic cholangiocarcinoma, HHLA2, PD-L1, Immunotherapy, Tumor associated macrophages, Tumor infiltrating lymphocytes, Prognosis

## Abstract

**Background:**

Intrahepatic cholangiocarcinoma (ICC) is a highly mortal malignancy with limited therapeutic options. Immunotherapies targeting PD-1/PD-L1 pathway represent a promising treatment for ICC. However, PD-L1 expression and microsatellite instability are not common in ICC. This study aimed to investigate whether HHLA2, a newly identified B7 family immune checkpoint for T cells, could be a therapeutic target next to PD-L1 in ICC.

**Methods:**

Expression levels of PD-L1 and HHLA2 as well as infiltrations of CD3+, CD8+, CD4 + Foxp3+, CD68+, CD163+ and CD20+ cells were evaluated by immunohistochemistry in 153 resected ICC samples. Comprehensive comparisons were made between PD-L1 and HHLA2 in terms of the expression rates, clinicopathological features and infiltrations of different immune cells. The expression level and prognostic significance of HHLA2 were further validated in an independent cohort.

**Results:**

Expression of HHLA2 is more frequent than PD-L1 in ICC (49.0% vs 28.1%). Co-expression of both immune checkpoints was infrequent (13.1%) and 50% PD-L1 negative cases were with elevated HHLA2. HHLA2 overexpression was associated with sparser CD3+ tumor infiltrating lymphocytes (TILs), CD8+ TILs and a higher CD4 + Foxp3+/CD8+ TIL ratio, whereas PD-L1 expression was associated with prominent T cells and CD163+ tumor associated macrophages infiltrations. PD-L1 failed to stratify overall survival (OS) but HHLA2 was identified as an independent prognostic indicator for OS in two independent cohorts.

**Conclusions:**

Compared with PD-L1, HHLA2 is more prevalent and possesses more explicit prognostic significance, which confer the rationale for HHLA2 as a potential immunotherapeutic target next to PD-L1 for ICC patients.

**Electronic supplementary material:**

The online version of this article (10.1186/s40425-019-0554-8) contains supplementary material, which is available to authorized users.

## Background

Intrahepatic cholangiocarcinoma (ICC) is the second most common primary liver malignancy [1]. The survival rates of ICC remain stagnant, despite great progresses have been made on the molecular basis, diagnosis and treatment modalities [1, 2]. Surgical resection offers the only chance to cure, but most ICC patients are diagnosed at advanced clinical stages when only palliative treatments can be performed [1]. Some of these palliative treatments are proved to be effective, but their prolongations of survival are still unsatisfactory [1, 3, 4]. Consequently, therapeutic targets that can significantly improve the survival of ICC are urgently needed.

Cancer cells can express immune-inhibitory molecules innately or adaptively to evade immune attacks from the hosts [5]. Recently, immunotherapies that normalize immune responses in the tumor microenvironment (TME), particularly through targeting the program cell death (PD) pathway, have been proved to achieve high objective response rates in several refractory malignancies [5–7]. To date, anti-PD therapy has been approved by FDA with more than 10 cancer indications, and PD-L1 expression level in tumor samples is an important biomarker to predict treatment responses of anti-PD therapy [8].

Although the effect of anti-PD therapy in biliary tract cancers remains scarcely reported, a recent case report showed that PD-1 inhibitor pembrolizumab brought strong and durable control to an advanced cholangiocarcinoma case [9]. Moreover, previous studies reported that PD-L1 expression rates ranged from 17.7 to 72.2% in different ICC cohorts and T cell infiltrates were found in majority of ICC samples [10–12]. These results altogether suggest that ICC is very likely to benefit from immunotherapies that normalize the TME. However, previous studies evaluating PD-L1 expression levels in ICC were different in materials, sample sizes and scoring systems. Gani reported that 39 out of 54 (72.2%) ICC cases were PD-L1 positive on cells within tumor front, whereas other studies mainly evaluated PD-L1 expression within tumor area and reported much lower expression rates ranging from 17.7 to 29.8% [10, 12]. Biomarkers that predict treatment responses towards anti-PD therapy are not limited to PD-L1 expression levels in tumor samples [8]. A PD-L1 negative cholangiocarcinoma with high-level microsatellite instability (MSI-H) were proved to be sensitive to anti-PD-1 therapy, which indicated that MSI status may also serve as a predictive biomarker for anti-PD therapy in ICC [9]. To our disappointment, the incidence of MSI-H in cholangiocarcinoma is also extremely low (1.3%, 4/308). Therefore, expression levels of other immune checkpoints are worth exploration to offer more immunotherapeutic choices for ICC patients who have been excluded from anti-PD therapy [9, 13].

HHLA2, short for HERV-HLTR-associating 2, is a newly identified immune checkpoint belonging to the B7 family [14]. Compared to other members of the B7 family, HHLA2 possesses some unique features: as a type I transmembrane molecule, it has three extracellular Ig domains [15]; it is constitutively expressed on peripheral monocytes and is inducible on B cells rather than on T cells [14, 15]. HHLA2 has been evidenced to inhibit TCR mediated proliferation and cytokine production of CD4+ and CD8+ T cells [14, 16]. Receptors of HHLA2 exist on a wide range of immune cells, such as T cells, monocytes and B cells, as well as on endothelial cells [15]. Most of these receptors remain unidentified, except for TMIGD2, a CD28 family member which is expressed on endothelial cells and may be a participant in angiogenesis [17, 18].

The expression pattern and clinical relevance of HHLA2 have so far been studied in detail in triple negative breast cancer (TNBC), osteosarcoma and non-small-cell lung cancer (NSCLC) [16, 17, 19, 20]. In TNBC and osteosarcoma, the high expression rates of HHLA2 were 56% (28/50) and 68% (42/62), respectively. Moreover, patients with high HHLA2 expression were more likely to have advanced and metastatic diseases [17, 19]. In NSCLC, 66% cases were HHLA2 positive (413/625). HHLA2 expression was associated with EGFR mutation status and high tumor infiltrating lymphocytes (TILs) density [20]. Moreover, HHLA2 was widely expressed in PD-L1 negative NSCLC samples [16]. These literature altogether indicated that HHLA2 was potentially involved in cancer progression through immune inhibition and could be a promising target next to PD-L1 for cancer immunotherapy [17, 19].

So far as we know, the expression pattern of HHLA2 and its association with PD-L1 expression in ICC remain unclear. In the present study, we performed a comprehensive comparison between HHLA2 and PD-L1 in terms of the expression pattern, clinical relevance and their associations with tumor infiltrating CD3+, CD8+, CD4 + Foxp3+, CD68+, CD163+ and CD20+ immune cells in a ICC cohort after curative resection.

## Methods

### Patient selection and follow-up procedures

Training cohort and validation cohort were derived from consecutive patients underwent curative resection for ICC from 2005 to 2014 in Zhongshan Hospital, Fudan University. All enrolled patients met the criteria as follows: (1) pathologically confirmed ICC; (2) received no anti-cancer treatments before surgical resection; (3) no history and concurrence of other malignant tumors; (4) complete removal of macroscopic tumors and negative resection margin proved by pathological examination; (5) complete clinicopathological and follow-up data.

The training and validation cohorts comprise 153 and 65 patients, respectively. Since the validation cohort was originally formed to study the mechanism of lymph node (LN) metastasis in ICC, it contains a higher proportion of patients with LN metastasis (46.2%). The baseline characteristics of the training and validation cohort are detailed in Table 1. Conventional serological tests, including CA19–9, carcino-embryonic antigen (CEA) levels were performed within 3 days before operation. Liver function was defined by albumin-bilirubin grade [21]. The clinical stage was determined by the American Joint Committee on Cancer (AJCC) 8th edition [22].

**Table 1:** Correlation between HHLA2 expression and baseline clinicopathological features in the training and validation cohort with ICC

Characteristics	Training cohort (*n* = 153)					Validation cohort (*n* = 65)				
	Patients		HHLA2 expression			Patients		HHLA2 expression		
	NO.	%	Low	High	*P-value*	NO.	%	Low	High	*P-value*
All patients	153	100.0	78	75		65	100.0	21	44	
Age					0.805					0.601
≤ 60	78	51.0	39	39		31	47.7	11	20	
> 60	75	49.0	39	36		34	52.3	10	24	
Liver cirrhosis					0.199					0.486
Absent	131	85.6	64	67		56	86.2	19	37	
Present	22	14.4	14	8		9	13.8	2	7	
ALBI grade					0.468					0.194
1	122	79.7	64	58		39	60.0	15	24	
2	31	20.3	14	17		26	40.0	6	20	
Tumor size					0.594					0.575
≤ 5 cm	64	41.8	31	33		37	56.9	13	24	
> 5 cm	89	58.2	47	42		28	43.1	8	20	
Tumor differentiation					0.833					0.368
I - II	126	82.4	65	61		30	46.2	8	22	
III - IV	27	17.6	13	14		35	53.8	13	22	
Tumor number					0.885					0.894
Single	113	73.9	58	55		52	80.0	17	35	
Multiple	40	26.1	20	20		13	20.0	4	9	
MVI					0.066					0.119
Absent	116	75.8	64	52		53	81.5	19	34	
Present	37	24.2	14	23		12	18.5	2	10	
LN metastasis					0.071					**0.013**
Absent	134	87.6	72	62		35	53.8	16	19	
Present	19	12.4	6	13		30	46.2	5	25	
CA19–9^a^					**< 0.001**					0.078
≤ 37 U/L	69	46.3	46	23		30	46.2	13	17	
> 37 U/L	80	53.7	30	50		35	53.8	8	27	
CEA^a^					**< 0.001**					**0.001**
≤ 5 ng/ml	121	81.2	72	49		36	55.4	18	18	
> 5 ng/ml	28	18.8	4	24		29	44.6	3	26	
AJCC 8th					0.053					**0.024**
I-II	122	79.7	67	55		19	29.2	10	9	
IIIa-IIIb	31	20.3	11	20		46	70.8	11	35	

Abbreviations: *ALBI* albumin-bilirubin, *MVI* microvascular invasion, *LN* lymph node, *CEA* carcinoembryonic antigen, *AJCC* American Joint Committee on Cancer; *P*-value < 0.05 marked in bold font shows statistical significant. ^a^For 4 patients of the training cohort, the data of CA19–9 and CEA were not available

Post-operative surveillance was performed as described in our previous study [23]. Overall survival (OS) was defined as the span from resection to death. Recurrence-free survival (RFS) was calculated from date of surgery to the day when recurrence was identified. For patients without OS/RFS event, the follow-up time was censored at the last follow-up. The last follow-up of all enrolled patients was censored at May 31st 2017. The median follow-up time of the whole studied population was 47.5 months (range 1–88.4).

### Tissue microarray (TMA), immunohistochemistry (IHC) and immunofluorescence (IF)

To make TMAs, the sections from formalin-fixed, paraffin-embedded surgical specimens were HE stained for selection of representative areas of tumor. Duplicate cores of 1 mm diameter were representative of tumor from each individual.

To perform IHC and IF, mouse monoclonal antibodies of CD3, CD8 (Abcam, Cambridge, UK), Foxp3 (R&D, Minneapolis, USA), rabbit monoclonal antibody of PD-L1 (clone E1L3N), CD68, CD163 (CST, Danvers, USA) as well as rabbit polyclonal antibodies of HHLA2 (Atlas, Stockholm, Sweden), CD4 (Maxim, Fuzhou, China), CD20 (Servicebio, Wuhan, China) were purchased.

IHC staining of TMA was performed as described in our previous study [24]. Briefly, slides were baked, deparaffinized and rehydrated. After blocking endogenous peroxidase activity in 0.3% H_2_O_2_, antigen retrieval was performed using EDTA buffer (PH 9.0) in microwave. None-specific binding sites were blocked by Protein block (Novocastra, Newcastle, UK) before antibody was incubated.

To perform IF, slides were prepared in the same manner as for IHC before incubation of antibodies. The incubation of CD4 antibody was followed by an anti-rabbit Alexa Fluor 594-conjugated secondary antibody. Subsequently, the slides were incubated in Foxp3 primary antibody and ensuing a second incubation in anti-mouse Alexa Fluor 488-conjugated secondary antibody (TermoFisher Scientifc).

### Quantification of HHLA2 and infiltration of T cells

The panoramas of IHC and IF staining of all slides were scanned and evaluated through Pannoramic Viewer (3DHISTECH, Budapest, HUNGARY). Slides were evaluated by 2 investigators under the guidance of a pathologist and without reference to clinical profiles of patients. Discrepant results between investigators were reconsidered and resolved together.

The immune staining density of HHLA2 was semi-quantified by H-score with the assistance of Densitoquant module from 3DHISTECH. As described in previous study, the H-score was generated by multiplying the percentage of immunoreactive cells by their corresponding staining intensity [20]. The expression of PD-L1 on tumor cells (TC) and immune cells (IC) were evaluated separately, PD-L1 staining on ≥5% tumor cells (TC) or PD-L1 stained immune cells (IC) ≥1% tumor area was defined as positive [25].

CD3+, CD8+, CD4 + Foxp3+, CD20+ TILs and CD68+ and CD163+ tumor associated macrophages (TAMs) were calculated in the same manner as described in our previous study [26, 27]. In brief, for each patient, the average of five independent microscopic fields (400×), which represented the densest lymphocytic infiltrates was used to reflect the extent of T cell infiltration. The cut-off values of all immune stained markers were determined by X-tile (New Haven, CT, USA) for optimal survival separation. Limited to the number of TMAs of the validation cohort, the evaluation of different types of TILs, TAMs as well as PD-L1 expression was only performed in training cohort.

### Statistical analysis

Associations between HHLA2, PD-L1 and other variables including densities of different subtypes of TILs, TAMs were evaluated using Chi-squared test, Fisher’s exact test or Mann-Whitney U test as appropriate. The survival curves of OS for patients with different HHLA2 and PD-L1 expression levels were depicted by Kaplan-Meier method and compared via the log-rank test. Univariate and multivariate analyses were performed based on Cox proportional hazard model. All statistical procedures were accomplished by SPSS version 21.0 (Chicago, IL, USA), Graphpad Prism 6 software (La Jolla, CA, USA).

## Results

### Baseline characteristics of patients

As illustrated in Table 1, the validation cohort had a larger proportion of patients with LN metastasis, elevated CEA levels and advanced AJCC stage compared with the training cohort.

In the training cohort, the median OS time was 28.8 months (range 1.0–88.4). The median RFS time was 13.6 months (range 1.0–88.4). The 1-, 3- and 5- year OS rates were 71.9, 44.4 and 32.7%, respectively. The 1-, 3- and 5- year RFS rates were 52.6, 32.1 and 30.3%, respectively. In the validation cohort, the median OS and RFS time was 16 months (range 1.0–62.0) and 10.4 months (range 1.0–62.0), respectively. The 1-, 3- and 5- year OS and RFS rates were 62.0, 20.7, 20.7% and 41.4, 17.0, 14.5%, respectively.

### Expression pattern of PD-L1 and HHLA2 in ICC

**Fig. 1:**
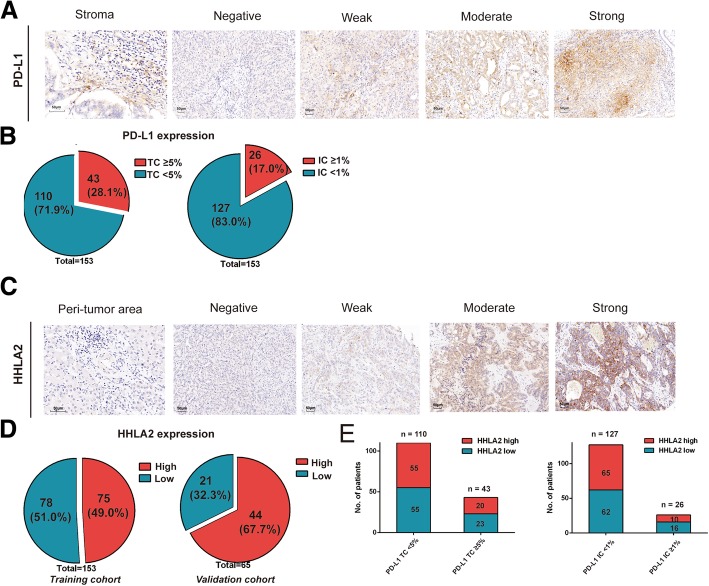
PD-L1 and HHLA2 expression in ICC tissue samples. Representative micrographs of PD-L1 (**a**) and HHLA2 (**c**) expression within tumor (scale bar, 50 μm). The positive rate of PD-L1 on tumor cells and immune cells were 28.1 and 17.0%, respectively (**b**). HHLA2 was elevated in 49.0 and 67.7% of cases in training and validation cohort, respectively (**d**). No significant correlation was found between HHLA2 and PD-L1 expression (**e**)

Typical microphotographs of PD-L1 expression are listed in Fig. 1a. The positive rate of PD-L1 on TC was 28.1% (43/153) (Fig. 1b). 17.0% (26/153) ICC patients was PD-L1 positive on IC (Fig. 1b).

As illustrated in Fig. 1c, negative to strong expression of HHLA2 was observed in ICC TMAs. The stained area was mostly in cytoplasm, on the membrane of tumor cells and the expression of HHLA2 was scarcely detected in para-tumor liver tissues, which were concordant with previous studies [17, 19, 20]. According to the calculation of X-tile, the optimal cut-off value for the H-score of HHLA2 expression was 5. Patients with H-score ≥ 5 was considered to be high HHLA2 expression and their counterparts with H-score < 5 were defined as low HHLA2 expression. In the training cohort, 51% (78/153) and 49.0% (75/153) of the patients were classified as low HHLA2 expression and high HHLA2 expression, respectively (Fig. 1d). The validation cohort comprised higher proportions of patients with high HHLA2 expression (training vs validation cohort, 49.0% vs 67.7%, *P* = 0.023; Fig. 1d).

No significant correlation was identified between PD-L1 and HHLA2 expression. In 13.1% (20/153) cases both immune checkpoints were detected. In PD-L1 TC- and IC- negative ICC, 50% (55/110) and 51.2% (65/127) patients were observed to express elevated HHLA2, respectively (Fig. 1e).

### Correlations between HHLA2, PD-L1 expression and clinical features

As detailed in Table 1, high HHLA2 expression was correlated with elevated serum CEA levels in both training and validation cohort (*P* ≤ 0.001 for both cohorts). Moreover, in the training cohort, patients with high HHLA2 were associated with abnormal CA19–9 levels (*P* < 0.023); in the validation cohort, HHLA2 overexpression had increased prevalence in patients with LN metastasis and advanced AJCC stage (*P* = 0.013 and 0.024, respectively).

Clinicopathological features of PD-L1 TC-positive and IC-positive patients are shown in Additional file 1: Table S1. PD-L1 positive on IC or TC was associated with fewer tumor nodules (*P* = 0.025 and *P* = 0.032, respectively). Moreover, PD-L1 positive on TC was associated with higher histological grade of the tumor (*P* = 0.037).

### Prognostic significances of HHLA2 and PD-L1

**Fig. 2:**
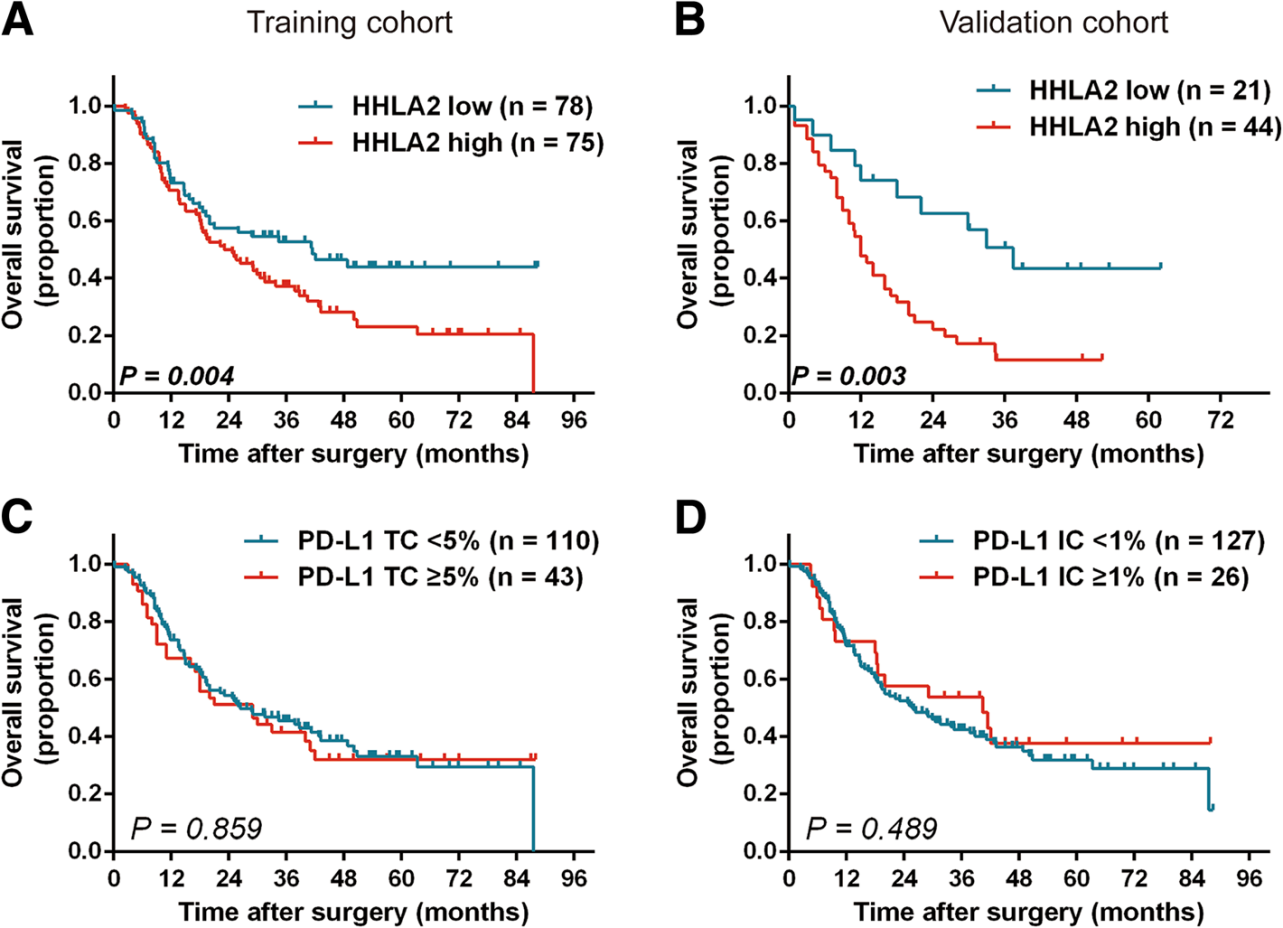
Kaplan Meier survival curves for OS of patients with ICC according to HHLA2 and PD-L1 expression. High HHLA2 expression was significantly associated with poor overall survival (OS) in the training cohort (**a**) and the significance was validated in an independent validation cohort (**b**). PD-L1 expression on TC (**c**) and IC (**d**) both failed to stratify OS in the training cohort. The *P*-values were determined via log-rank test

The results of univariate and multivariate analyses for OS were presented in Table 2. In univariate analysis for OS in the training cohort, tumor multiplicity (*P* < 0.001), microvascular invasion (MVI) (*P* = 0.001), LN metastasis (*P* < 0.001), elevated serum CA19–9 level (*P* = 0.048), high HHLA2 expression (*P* = 0.004; Fig. 2a) and advanced AJCC 8th stage (*P* < 0.001) were found to have a significant correlation with unfavorable OS. In multivariate analysis, tumor multiplicity (*P* = 0.001, hazard ratio [HR] = 2.167, 95%CI 1.375–3.416), MVI (*P* = 0.007, HR = 1.847, 95%CI 1.179–2.892), LN metastasis (*P* = 0.001, HR = 2.711 95%CI 1.534–4.791) and high HHLA2 expression (*P* = 0.025, HR = 1.593, 95%CI 1.059–2.396) continued to be prognostic indicators for OS.

**Table 2:** Univariate and multivariate analyses of prognostic factors correlated with OS

Variables	Overall survival					
	Training cohort (*n* = 153)			Validation cohort (*n* = 65)		
	Univariate *P*-value	Multivariate *P*-value	Multivariate HR (95%CI)	Univariate *P*-value	Multivariate *P*-value	Multivariate HR (95%CI)
Gender (male vs female)	0.300	NA	NA	0.501	NA	NA
Age, years (> 60 vs ≤ 60)	0.350	NA	NA	0.365	NA	NA
Liver cirrhosis (yes vs no)	0.209	NA	NA	0.077	NA	NA
ALBI grade (2 vs 1)	0.876	NA	NA	0.075	NA	NA
Tumor size, cm (> 5 cm vs ≤ 5)	0.231	NA	NA	0.137	NA	NA
Tumor number (multiple vs single)	**< 0.001**	**0.001**	2.167 (1.375–3.416)	0.584	NA	NA
MVI (yes vs no)	**0.001**	**0.007**	1.847 (1.179–2.892)	0.784	NA	NA
LN metastasis (yes vs no)	**< 0.001**	**0.001**	2.711 (1.534–4.791)	**0.004**	0.053	1.879 (0.993–3.577)
Tumor differentiation (III-IV vs I-II)	0.223	NA	NA	0.495	NA	NA
CA19–9,U/L (> 37 vs ≤ 37)	**0.048**	0.261	1.295 (0.825–2.032)	**0.001**	**0.009**	2.369 (1.242–4.519)
CEA, ng/ml (> 5 vs ≤ 5)	0.239	NA	NA	**0.001**	0.109	1.735 (0.885–3.401)
HHLA2 expression (high vs low)	**0.004**	**0.025**	1.593 (1.059–2.396)	**0.003**	**0.014**	2.459 (1.197–5.049)
PD-L1 expression (TC ≥5% vs TC < 5%)	0.859	NA	NA	NA	NA	NA
PD-L1 expression (IC ≥1% vs IC < 1%)	0.489	NA	NA	NA	NA	NA
AJCC 8th edition (IIIa-IIIb vs I-II)	**< 0.001**	NA	NA	0.120	NA	NA

Abbreviations: *HR* hazard ratio, *CI* confidence interval, *NA* not available, *ALBI* albumin-bilirubin, *MVI* microvascular invasion, *LN* lymph node, *CEA* carcinoembryonic antigen, *TC* tumor cells, *IC* immune cells, *AJCC* American Joint Committee on Cancer; Variables with strong correlations were not analyzed together in multivariate analyses to avoid confounded results. *P*-value < 0.05 marked in bold font shows statistical significant

In the validation cohort, which comprises more patients with LN metastasis, the presence of LN metastasis (*P* = 0.004), raised CEA (*P* = 0.001) and CA19–9 levels (*P* = 0.001) and overexpression of HHLA2 (*P* = 0.003; Fig. 2b) were found to stratify OS significantly. Whereas in multivariate analysis, only elevated CA19–9 level (*P* = 0.009, HR = 2.369, 95%CI 1.242–4.519) and high HHLA2 expression (*P* = 0.014, HR = 2.459, 95%CI 1.197–5.049) were identified as independent prognostic factors for OS.

In terms of RFS, multiple tumors (*P* < 0.001), MVI (*P* = 0.002) and LN metastasis (*P* = 0.013) were found to be prognostic indicators in both univariate analysis and multivariate analysis in the training cohort (Additional file 2: Table S2). High HHLA2 expression failed to stratify RFS for training cohort (*P* = 0.069).

To sum up, high HHLA2 expression was identified as an independent risk factor for OS in both training and validation cohort (Fig. 2a and b). PD-L1 failed to stratify OS (*P* = 0.859 and *P* = 0.489 for TC and IC expression, respectively; Fig. 2c and d; Table 2) and RFS (*P* = 0.781 and *P* = 0.063 for TC and IC expression, respectively; Additional file 2: Table S2) in the training cohort.

### Tumor infiltrating T cells and their associations with PD-L1 and HHLA2 expression

**Fig. 3:**
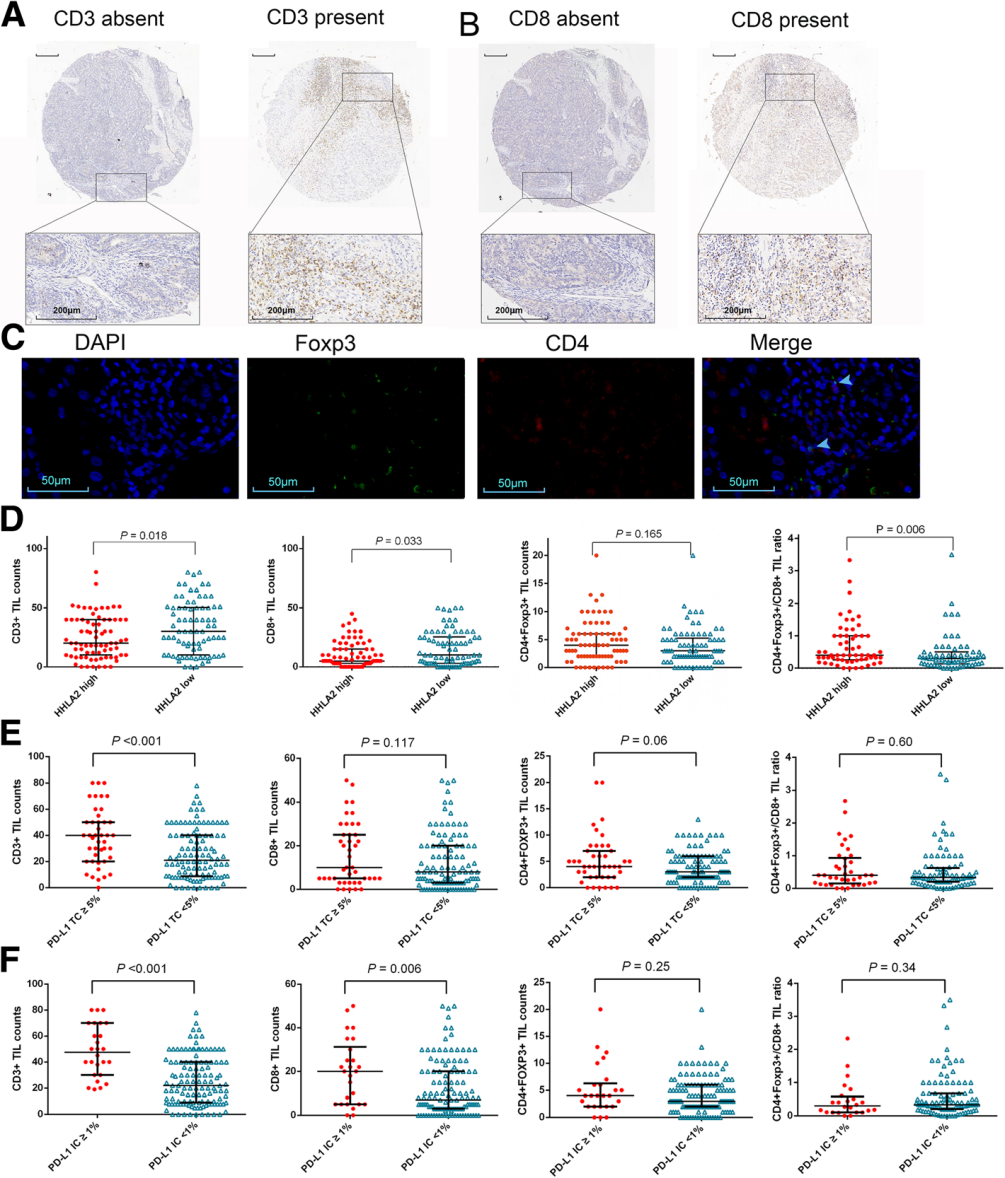
Tumor infiltrating T cells, cytotoxic T cells (CTLs) and regulatory T cells (Tregs) and their correlation between HHLA2 and PD-L1 expression. Images of positive CD3 (**a**), CD8 (**b**) staining and the corresponding intra-tumor negative controls. Magnification × 50 for full views and × 400 for zoomed-in views (scale bar, 200 μm). Cells with double staining of CD4 and Foxp3 were identified as Tregs (Arrow) (**c**). Original magnification × 500 (scale bar, 50 μm). Scatter plot depicted the correlation between classic subsets of T cells and HHLA2 expression (**d**). High HHLA2 expression was significantly correlated with lower intra-tumor counts of T cells and cytotoxic T cells as well as increased ratio of Tregs to CTLs. PD-L1 expression was associated with higher intra-tumor counts of T cells and cytotoxic T cells (**e** and **f**)*. P -*values were generated by Mann-Whitney U test. Error bars indicate median and interquartile range

Microphotographs of CD3+, CD8+ and CD4 + Foxp3+ TILs, which represent overall T cells, cytotoxic T cells (CTLs) and regulatory T cells (Tregs), respectively, are presented in Fig. 3. CD3+ TILs were observed in intratumoral area of 94.1% (144/153) patients in training cohort, which indicated a high prevalence of T cell infiltrates in ICC patients. In addition, CD8+ TILs and CD4 + Foxp3+ TILs were identified in 83.0% (127/153) and 86.9% (133/153) patients, respectively.

As detailed in Additional file 3: Table S3, high expression of HHLA2 was associated with lower intratumoral CD3+, CD8+ TIL counts (*P* = 0.018 and 0.033, respectively). No significant correlation was observed between the counts of CD4 + Foxp3+ T cells and HHLA2 expression. Intriguingly, when the ratio of CD4 + Foxp3+ TILs and CD8+ TILs was generated, we identified that high expression of HHLA2 was correlated with higher CD4 + Foxp3+/CD8+ TIL ratio (*P* = 0.006), which was indicative of an immune-inhibitory TME in patients with high HHLA2 expression (Fig. 3d).

In contrast to an immune-inhibitory TME found in ICC with elevated HHLA2, PD-L1 IC- and TC- positive cases were associated with prominent infiltration of CD3+ T cells (Fig. 3e and f; *P* < 0.001 for both comparisons). Moreover, PD-L1 expression on IC were correlated with higher CD8+ TILs counts (Fig. 3e; Additional file 4: Table S4 and Additional file 5: Table S5).

The prognostic significances of the variables concerning on the intratumoral infiltrations of different T cell subsets for ICC were evaluated via univariate analysis in the training cohort (Additional file 6: Table S6). High intratumoral counts of CD8+ TILs, high CD8+/CD3+ TIL ratio and low CD4 + Foxp3+/CD8+ ratio were found to be significantly associated with higher OS and RFS rates.

### Prevalence of TAMs and B cells and their correlations with PD-L1 and HHLA2

**Fig. 4:**
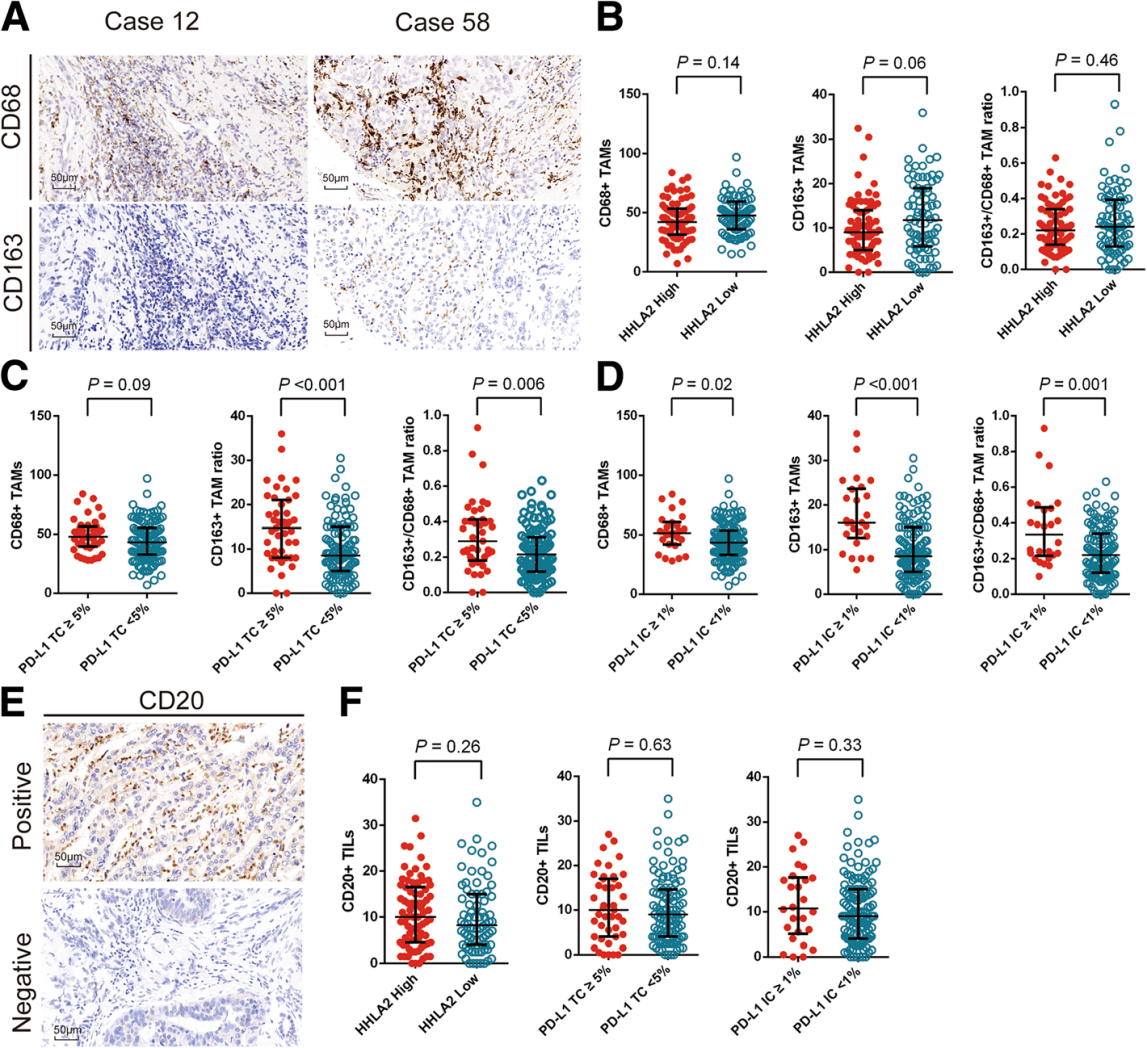
CD68+ tumor associated macrophages (TAMs), CD163+ TAMs and CD20+ tumor infiltrating lymphocytes (TILs) and their correlation between HHLA2 and PD-L1 expression. Representative images of CD68 and CD163 staining (**a**; scale bar, 50 μm). No significant differences were found on the infiltrations of CD68+ TAMs, CD163+ TAMs as well as CD163+/CD68+ TAMs ratio between ICC with different HHLA2 expression levels (**b**). PD-L1 expression on TC was significantly correlated with a higher density of CD163+ TAMs and a higher CD163+/CD68+ TAMs ratio (**c**). PD-L1 expression on IC was significantly correlated with prominent infiltrations of CD68+ TAMs and CD163+ TAMs as well as a higher CD163+/CD68+ TAMs ratio (**d**). Images of positive CD20 staining and the corresponding intra-tumor negative control (**e**; scale bar, 50 μm). No significant differences were found on CD20+ TIL counts between ICC with different HHLA2 and PD-L1 expression levels (**f**). *P -*values were generated by Mann-Whitney U test. Error bars indicate median and interquartile range

Microphotographs of CD68+ and CD163+ TAMs are shown in Fig. 4a. CD163 is mainly expressed M2-like macrophages and CD68 is positive on all macrophages [28]. CD68+ TAMs were found in all ICC samples and CD163+ TAMs were detected in 96.7% (148/153) of the ICC samples.

As illustrated in Fig. 4b, no significant differences were found on the counts of CD68+ (*P* = 0.14), CD163+ (*P* = 0.06) TAMs as well as the ratio of CD163+ TAMs to CD68 + TAMs (*P* = 0.46) between patients with different HHLA2 expression levels (Additional file 3: Table S3).

PD-L1 expression on TC was positively correlated with CD163+ TAM counts (*P* < 0.001) and CD163+/CD68+ TAM ratio (*P* = 0.006; Fig. 4c). PD-L1 expression on IC was positively associated with CD68+ (*P* = 0.02), CD163+ (*P* < 0.001) TAM counts as well as the CD163+/CD68+ TAM ratio (*P* = 0.001; Fig. 4d; Additional file 4: Table S4 and Additional file 5: Table S5).

Representative images of CD20 staining are shown in Fig. 4e. CD20+ TILs, which indicate the infiltration of B cells, were observed in 94.8% (145/153) of the ICC patients. However, there are no significant differences in the distribution of CD20+ TILs between patients with different levels of HHLA2 (*P* = 0.26) and PD-L1 (*P* = 0.63 and *P* = 0.33 for TC and IC expression, respectively; Fig. 4f). Unlike tumor infiltrating T cells, CD68+, CD163+ TAMs and CD20+ TILs all failed to be prognostic indicators for OS and RFS (Additional file 6: Table S6).

## Discussion

In this study, we observed that HHLA2, a newly identified B7 family member, was more prevalent than PD-L1 in ICC and HHLA2 overexpression was common in PD-L1 negative ICC. Moreover, HHLA2 was identified as an independent prognostic indicator for OS in two independent cohorts whereas PD-L1 showed no significant prognostic value. The presence of PD-L1 and HHLA2 was associated with differential infiltration of immune cells: PD-L1 positive tumors were observed to have higher densities of CD3 + TILs, CD68+ TAMs and CD163+ TAMs, whereas HHLA2 overexpression was significantly correlated with sparser CD3+ TILs, CD8+ TILs and a higher CD4 + Foxp3+/CD8+ TIL ratio.

HHLA2 was previously observed with expression rates ranging from zero to 70% in a variety of cancer types [17]. The high expression rates of HHLA2 in the two independent ICC cohorts were 49.0 and 67.7% respectively, which was on a medium to high end of the expression spectrum, higher than previous data on gall bladder cancers (0/10) and liver cancers (4/10) [17]. We found that HHLA2 expression was positively associated with serum CEA and CA19–9 levels in the training cohort as well as the presence of LN metastasis, serum CEA and clinical stage in the validation cohort. In addition, in the validation cohort which was featured by more LN metastases, HHLA2 expression was more frequently observed. These data were in line with previous findings in TNBC and osteosarcoma, where HHLA2 expression was found to be more frequent in patients with LN metastasis and metastatic lesions [17, 19]. Moreover, we found that HHLA2 expression strongly predicted poor OS in the two independent ICC cohorts. The basis for these results may, on one hand, attributed to the known co-inhibitory roles of HHLA2 on T cells [14]. On the other hand, the positive correlations between HHLA2 expression and serum CEA, CA19–9 levels, two tumor biomarkers that generally reflect tumor burden both at primary sites and in the circulation for ICC, indicating that patients with HHLA2 overexpression are more likely to suffer from recurrence and metastasis [29]. Along with previous findings, in ICC, HHLA2 may not only work as an inhibitory checkpoint, but also potentially contribute to tumor progression through binding to TMIGD2, a recently identified ligand which is also involved in angiogenesis, or through other unknown mechanisms [18]. Therefore, targeting HHLA2 may inhibit cancer dissemination through immune-independent pathways and the underlying molecular basis requires further investigation.

Our study reported a PD-L1 positive rate of 28.1 and 17.1% on TC and IC, respectively. These results were similar to a previous report from another large Asian ICC cohort (*n* = 192), in which 17.7% of the cases were found to be PD-L1 positive [12]. Intriguingly, the prognostic significances of PD-L1 were inconsistent among different studies [10–12]. Studies of Gani et al. and Sabbatino et al. observed that PD-L1 positive was associated with unfavorable survival, but Zhu et al. reported opposite results [10–12]. In our study, PD-L1 expression had no significant correlation with survival. Moreover, we identified that PD-L1 positive cases had prominent T cell infiltration, which was similar to findings from Zhu et al. in ICC and the report from Schalper et al. concerning lung cancer [30]. The infiltration of T cells, especially CD8+ T cells, is a known factor that indicates favorable survival and this finding was also confirmed in our cohort (Additional file 6: Table S6). Although PD-L1 expression, based on the molecular basis, is speculated to have a negative impact on survival, whereas its positive correlation with T cell infiltration mainly exert an opposing impact and consequently leads to the uncertainty of prognostic significance [24]. However, the discrepant prognostic significances of PD-L1 may not be a limitation for its possible role as a therapeutic target and a predictive biomarker for treatment response [30].

Previous studies reported discrepant results among different cancer types on the correlations between immune cell infiltration and HHLA2 expression. In NSCLC, HHLA2 expression was independently correlated with high TIL infiltration [20]. Whereas for osteosarcoma, no significant correlation was found between the presence of TILs and HHLA2 expression [19]. In this study, we evaluated the immune cells in a more detailed manner where the exact counts of stained immune cells of 5 high power field were calculated [24]. We observed that HHLA2 overexpression was conversely associated with T cell and CTL infiltration in ICC. Although no significant correlation was found between HHLA2 expression and the counts of Tregs, patients with high HHLA2 expression were found to have higher ratio of CTLs to Tregs within tumor area. Collectively speaking, the expression of HHLA2 was correlated with an inhibitory TME featured by decreased T cells, CTLs and imbalance between Tregs and CTLs, which indicated the possible role of HHLA2 as an immunotherapeutic target.

HHLA2-high patients and PD-L1 positive cases showed significant difference in the infiltrating patterns of immune cells. HHLA2 was associated with fewer CTLs and higher ratio of Treg to CTLs, whereas the presence of PD-L1 was accompanied by prominent T cells and CD163+ TAMs. This phenomenon is intricate but explainable. The positive correlation between T cell infiltration and PD-L1 expression on TC may indicate that, in ICC, PD-L1 is mainly adaptively expressed in tumor immune response, perhaps through the well-established IFN-γdependent manner [31]. CD163+ TAMs mainly represent M2-like TAMs which contribute to immune suppression [32]. TAMs have been observed to express PD-L1 and induce immune evasion of cancer through autocrine CXCL8 [33]. Moreover, targeting PD-L1 can polarize macrophages to a more pro-inflammatory phenotype [34]. Therefore, the significant infiltration of CD163+ TAMs in PD-L1 IC- and TC- positive ICC may also be a consequence of immune reactions to tumor. On the other hand, concerning the high expression rate of HHLA2 and its correlation with sparser T cell infiltration, HHLA2 is more likely to be innately expressed in ICC and thus limiting further infiltration of active T cells. Although HHLA2 can be induced on B cells and monocytes by IFN-γ and LPS, the mechanism of how tumor cells enhance HHLA2 expression remains to be further investigated [14, 17].

Several limitations concerning this study were underlined as follows. Firstly, both training and validation cohort were derived from a single institution in China, therefore the expression pattern of HHLA2 of ICC patients in other ethnic groups has yet to be investigated. Secondly, limited to the number of TMAs, PD-L1 expression as well as the correlations between typical subsets of tumor infiltrating immune cells and PD-L1, HHLA2 expression were only studied in our training cohort. Therefore, validation on the immune infiltration features of ICC with PD-L1, HHLA2 expression in another independent ICC cohort as well as further investigation on its molecular basis will provide more solid evidence.

## Conclusions

In summary, HHLA2 is more commonly expressed and possesses more significant prognostic value compared with PD-L1 in ICC cases. High HHLA2 is frequent in PD-L1 negative cases and is associated with fewer infiltrating CTLs as well as more Tregs in proportion, suggesting that HHLA2 may represent an ideal target next to PD-L1 for immunotherapy of ICC.

## Additional files

Additional file 1: **Table S1.** Correlation between PD-L1 expression and baseline clinicopathological features in ICC (DOCX 19 kb)

Additional file 2: **Table S2.** Univariate and multivariate analyses of prognostic factors correlated with RFS (DOCX 16 kb)

Additional file 3: **Table S3.** Correlation between HHLA2 expression and different immune infiltrates (DOCX 16 kb)

Additional file 4: **Table S4.** Correlation between PD-L1 expression on TC and different immune infiltrates (DOCX 16 kb)

Additional file 5: **Table S5.** Infiltrating patterns of immune cells with different PD-L1 expression on IC (DOCX 16 kb)

Additional file 6: **Table S6.** Univariate analyses of densities of different immune cells for OS and RFS in ICC. (DOCX 17 kb)
