# Supplementary material for: HHLA2 in intrahepatic cholangiocarcinoma: an immune checkpoint with prognostic significance and wider expression compared with PD-L1
Source: J Immunother Cancer. 2019 Mar 18;7:77. doi: 10.1186/s40425-019-0554-8 (PMC6421676; doi:10.1186/s40425-019-0554-8)
Supplement: Supplementary file 1 — Table S1. Correlation between PD-L1 expression and baseline clinicopathological features in ICC (DOCX 19 kb) [file 40425_2019_554_MOESM1_ESM.docx]

| **Table S1. Correlation between PD-L1 expression and baseline clinicopathological features in ICC.** | | | | | | | | |
| --- | --- | --- | --- | --- | --- | --- | --- | --- |
| **Characteristics** | **Training cohort (n = 153)** | | | | | | | |
|  |  | **IC <1%**  **n (%)** | **IC ≥1%**  **n (%)** | ***P-value*** |  | **TC <5%**  **n (%)** | **TC ≥5%**  **n (%)** | ***P-value*** |
| All patients |  | 127 (83.0) | 26 (17.0) |  |  | 110 (71.9) | 43 (28.1) |  |
| Age |  |  |  | 0.452 |  |  |  | 0.455 |
| ≤ 60 |  | 63 (49.6) | 15 (57.7) |  |  | 54 (49.1) | 24 (55.8) |  |
| > 60 |  | 60 (50.4) | 11 (42.3) |  |  | 56 (50.9 ) | 19 (44.2) |  |
| Liver cirrhosis |  |  |  | 0.286 |  |  |  | 0.544 |
| Absent |  | 107 (84.3) | 24 (92.3) |  |  | 93 (84.5) | 38 (55.8) |  |
| Present |  | 20 (15.7) | 2 (7.7) |  |  | 17 (15.5) | 5 (44.2） |  |
| ALBI grade |  |  |  | 0.290 |  |  |  | 0.405 |
| 1 |  | 98 (77.2) | 23 (88.5) |  |  | 88 (80.0) | 33 (76.7) |  |
| 2 |  | 29 (22.8) | 3 (11.5) |  |  | 22 (20.0) | 10 (23.3) |  |
| Tumor size |  |  |  | 0.173 |  |  |  | 0.712 |
| ≤ 5cm |  | 50 (39.4) | 14 (53.8) |  |  | 45 (40.9) | 19 (44.2) |  |
| > 5cm |  | 66 (60.6) | 12 (42.6) |  |  | 65 (59.1) | 24 (55.8) |  |
| Tumor differentiation |  |  |  | 0.425 |  |  |  | **0.037** |
| I - II |  | 106 (83.5) | 20 (76.9) |  |  | 95 (86.4) | 31 (72.1) |  |
| III - IV |  | 21 (16.5) | 6 (23.1) |  |  | 15 (13.6) | 12 (27.9) |  |
| Tumor number |  |  |  | **0.025** |  |  |  | **0.032** |
| Single |  | 89 (70.1) | 24 (92.3) |  |  | 76 (69.1) | 37 (86.0) |  |
| Multiple |  | 38 (29.9) | 2 (7.7) |  |  | 34 (30.9) | 6 (14.0) |  |
| MVI |  |  |  | 0.720 |  |  |  | 0.867 |
| Absent |  | 97 (76.4) | 19 (73.1) |  |  | 83 (75.5) | 33 (86.0) |  |
| Present |  | 30 (23.6) | 7 (26.9) |  |  | 27 (24.5) | 10 (23.3) |  |
| LN metastasis |  |  |  | 0.533 |  |  |  | 0.719 |
| Absent |  | 110 (86.6) | 24 (92.3) |  |  | 97 (88.2) | 37 (86.0) |  |
| Present |  | 17 (13.4) | 2 (7.7) |  |  | 13 (11.8) | 6 (14.0) |  |
| CA19-9* |  |  |  | 0.853 |  |  |  | 0.208 |
| ≤ 37U/L |  | 57 (46.0) | 12 (48.0) |  |  | 53 (49.5) | 16 (38.1) |  |
| > 37U/L |  | 67 (54.0) | 13 (52.0) |  |  | 54 (50.5) | 26 (61.9) |  |
| CEA* |  |  |  | 0.695 |  |  |  | 0.148 |
| ≤ 5ng/ml |  | 100 (80.6) | 21 (84.0) |  |  | 90 (84.1) | 31 (73.8) |  |
| > 5ng/ml |  | 24 (19.4) | 4 (16.0) |  |  | 17 (15.9) | 11 (26.2) |  |
| AJCC 8th |  |  |  | 0.885 |  |  |  | 0.306 |
| I-II |  | 101 (79.5) | 21 (80.8) |  |  | 90 (81.8) | 32 (74.4) |  |
| IIIa-IIIb |  | 26 (20.5) | 5 (19.2) |  |  | 20 (18.2) | 11 (25.6) |  |
| Abbreviations: ALBI, albumin-bilirubin; MVI, microvascular invasion; LN, lymph node; CEA, carcinoembryonic antigen; IC, immune cells; TC, tumor cells; AJCC, American Joint Committee on Cancer; P-value < 0.05 marked in bold font shows statistical significant. *For 4 patients of the training cohort, the data of CA19-9 and CEA were not available. | | | | | | | | |
