# Supplementary material for: HHLA2 in intrahepatic cholangiocarcinoma: an immune checkpoint with prognostic significance and wider expression compared with PD-L1
Source: J Immunother Cancer. 2019 Mar 18;7:77. doi: 10.1186/s40425-019-0554-8 (PMC6421676; doi:10.1186/s40425-019-0554-8)
Supplement: Supplementary file 2 — Table S2. Univariate and multivariate analyses of prognostic factors correlated with RFS (DOCX 16 kb) [file 40425_2019_554_MOESM2_ESM.docx]

| **Table S2. Univariate and multivariate analyses of prognostic factors correlated with RFS.** | | | | | | | | |
| --- | --- | --- | --- | --- | --- | --- | --- | --- |
| **Variables** | **Recurrence-free survival** | | | | | | |  |
|  | **Training cohort (n = 153)** | | |  | **Validation cohort (n = 65)** | | |  |
|  | **Univariate *P*-value** | **Multivariate *P*-value** | **Multivariate HR (95%CI)** |  | **Univariate *P*-value** | **Multivariate *P*-value** | **Multivariate HR (95%CI)** |  |
| Gender, male/female | 0.566 | NA | NA |  | 0.181 | NA | NA |  |
| Age, years ( > 60 vs ≤ 60) | 0.995 | NA | NA |  | 0.888 | NA | NA |  |
| Liver cirrhosis (yes vs no) | 0.680 | NA | NA |  | 0.228 | NA | NA |  |
| ALBI grade (2 vs 1) | 0.368 | NA | NA |  | 0.554 | NA | NA |  |
| Tumor size, cm (> 5cm vs ≤ 5) | 0.181 | NA | NA |  | 0.110 | NA | NA |  |
| Tumor number (multiple vs single) | **<0.001** | **<0.001** | 2.235 (1.414 - 3.484) |  | 0.869 | NA | NA |  |
| MVI (yes vs no) | **0.002** | **0.002** | 2.027 (1.295 - 3.173) |  | 0.905 | NA | NA |  |
| LN metastasis (yes vs no) | **0.001** | **0.013** | 2.057 (1.167 - 3.626) |  | **0.023** | 0.074 | 1.672 (0.951 - 2.940) |  |
| Tumor differentiation (III-IV vs I-II) | 0.781 | NA | NA |  | 0.168 | NA | NA |  |
| CA19-9,U/L (> 37 vs ≤ 37） | 0.398 | NA | NA |  | 0.104 | NA | NA |  |
| CEA, ng/ml (> 5 vs ≤ 5） | 0.916 | NA | NA |  | **0.043** | 0.278 | 1.393 (0.765 - 2.538) |  |
| HHLA2 expression (high vs low） | 0.069 | NA | NA |  | **0.016** | **0.016** | 2.252 (1.161 - 4.368) |  |
| PD-L1 expression (TC ≥5% vs TC <5%) | 0.781 | NA | NA |  | NA | NA | NA |  |
| PD-L1 expression (IC ≥1% vs IC <1%) | 0.063 | NA | NA |  | NA | NA | NA |  |
| AJCC 8th edition (IIIa-IIIb vs I-II) | **0.029** | NA | NA |  | 0.836 | NA | NA |  |
| Abbreviations: HR, hazard ratio; CI, confidence interval; NA, not available; ALBI, albumin-bilirubin; MVI, microvascular invasion; LN, lymph node; AFP, α-fetoprotein; CEA, carcinoembryonic antigen; TC, tumor cells; IC, immune cells; AJCC, American Joint Committee on Cancer; P-value <0.05 marked in bold font shows statistical significant. | | | | | | | | |
