# Supplementary material for: HHLA2 in intrahepatic cholangiocarcinoma: an immune checkpoint with prognostic significance and wider expression compared with PD-L1
Source: J Immunother Cancer. 2019 Mar 18;7:77. doi: 10.1186/s40425-019-0554-8 (PMC6421676; doi:10.1186/s40425-019-0554-8)
Supplement: Supplementary file 3 — Table S3. Correlation between HHLA2 expression and different immune infiltrates (DOCX 16 kb) [file 40425_2019_554_MOESM3_ESM.docx]

| **Table S3. Correlation between HHLA2 expression and different immune infiltrates.** | | | |
| --- | --- | --- | --- |
| **Variables** | **HHLA2 expression** | | |
|  | **Low (n = 78)** | **High (n = 75)** | ***P-value*** |
| CD3+TILs (median, IQR) | 30, 10 - 50 | 20, 10 - 40 | **0.018** |
| CD8+TILs (median, IQR) | 10, 3 - 25.5 | 5, 3 - 15 | **0.033** |
| CD4+Foxp3+TILs (median, IQR) | 3, 2 - 5.25 | 4, 2 - 6 | 0.165 |
| CD8+/CD3+ TIL ratio* (median, IQR) | 0.50, 0.23 - 0.64 | 0.40, 0.23 - 0.60 | 0.387 |
| CD4+Foxp3+/CD8+ TIL ratio^Ψ^ (median, IQR) | 0.30, 0.13 - 0.50 | 0.40, 0.25 - 1.00 | **0.006** |
| CD68+ TAMs (median, IQR) | 47.5, 36.0 - 59.5 | 42.0, 31.5 - 53.5 | 0.14 |
| CD163+ TAMs (median, IQR) | 11.75, 5.80 - 19.0 | 9.0, 5.0 - 14.0 | 0.06 |
| CD163+/CD68+ TAM ratio (median, IQR) | 0.239, 0.130 -0.396 | 0.221, 0.145 - 0.339 | 0.46 |
| CD20+ TILs (median, IQR) | 8.25, 4.0 - 15.0 | 10.0, 4.5 - 16.5 | 0.26 |
| CD3+TIL |  |  | **0.007** |
| < 50 | 52 | 64 |  |
| ≥ 50 | 26 | 11 |  |
| CD8+TILs |  |  | 0.332 |
| < 5 | 11 | 15 |  |
| ≥ 5 | 67 | 60 |  |
| CD4+Foxp3+TILs |  |  | 0.092 |
| < 8 | 70 | 60 |  |
| ≥ 8 | 8 | 15 |  |
| CD8+/CD3+ TILs ratio* |  |  | 0.518 |
| ≤ 0.4 | 34 | 35 |  |
| > 0.4 | 41 | 34 |  |
| CD4+Foxp3+/CD8+ TILs ratio^Ψ^ |  |  | **0.03** |
| ≤ 0.4 | 54 | 38 |  |
| > 0.4 | 13 | 22 |  |

Abbreviations: IQR, interquartile range; TIL, tumor infiltrating lymphocytes. TAM, tumor associated macrophages. *The CD8+/CD3+ TILs ratio was not applicable in 9 patients with no CD3+ TILs. ^Ψ^ The CD4+Foxp3+/CD8+ TILs ratio was not applicable in 26 patients with no CD8+ TILs ratio.
