# Supplementary material for: HHLA2 in intrahepatic cholangiocarcinoma: an immune checkpoint with prognostic significance and wider expression compared with PD-L1
Source: J Immunother Cancer. 2019 Mar 18;7:77. doi: 10.1186/s40425-019-0554-8 (PMC6421676; doi:10.1186/s40425-019-0554-8)
Supplement: Supplementary file 4 — Table S4. Correlation between PD-L1 expression on TC and different immune infiltrates (DOCX 16 kb) [file 40425_2019_554_MOESM4_ESM.docx]

| **Table S4. Correlation between PD-L1 expression on TC and different immune infiltrates.** | | | |
| --- | --- | --- | --- |
| **Variables** | **PD-L1 expression** | | |
|  | **TC <5%**  **(n = 110)** | **TC ≥5%**  **(n = 43)** | ***P-value*** |
| CD3+TILs (median, IQR) | 21 , 8.75 – 40 | 40 ,20 - 50 | **0.001** |
| CD8+TILs (median, IQR) | 8 , 3 - 20 | 10 , 5 - 25 | 0.117 |
| CD4+Foxp3+TILs (median, IQR) | 3 , 2 - 6 | 4 , 2 - 7 | 0.065 |
| CD8+/CD3+ TILs ratio* (median, IQR) | 0.4, 0.2 - 0.6 | 0.4, 0.2 - 0.6 | 0.328 |
| CD4+Foxp3+/CD8+ TILs ratio^Ψ^ (median, IQR) | 0.3, 0.2 - 0.6 | 0.4,0.2 - 0.9 | 0.617 |
| CD68+ TAMs (median, IQR) | 42.25, 32.75 - 55.38 | 48.0, 39.5- 56.5 | 0.087 |
| CD163+ TAMs (median, IQR) | 8.5, 5.0 -15.0 | 14.67, 8.0 - 21.0 | **0.003** |
| CD163+/CD68+ TAM ratio (median, IQR) | 0.22, 0.12 - 0.31 | 0.29, 0.18 - 0.41 | **0.006** |
| CD20+ TILs (median, IQR) | 9.0, 4.0 - 14.6 | 10.0, 4.0 - 17.0 | 0.634 |
| CD3+TIL |  |  | 0.053 |
| < 50 | 88 | 28 |  |
| ≥ 50 | 22 | 15 |  |
| CD8+TILs |  |  | 0.269 |
| < 5 | 21 | 5 |  |
| ≥ 5 | 89 | 38 |  |
| CD4+Foxp3+TILs |  |  | 0.377 |
| < 8 | 100 | 37 |  |
| ≥ 8 | 10 | 6 |  |
| CD8+/CD3+ TILs ratio* |  |  | 0.491 |
| ≤ 0.4 | 47 | 22 |  |
| > 0.4 | 55 | 20 |  |
| CD4+Foxp3+/CD8+ TILs ratio^Ψ^ |  |  | 0.273 |
| ≤ 0.6 | 67 | 25 |  |
| > 0.6 | 22 | 13 |  |

Abbreviations: IQR, interquartile range; TIL, tumor infiltrating lymphocytes; TAM, tumor associated macrophages. TC, tumor cells. * The CD8+/CD3+ TILs ratio was not applicable in 9 patients with no CD3+ TILs. ^Ψ^ The CD4+Foxp3+/CD8+ TILs ratio was not applicable in 26 patients with no CD8+ TILs ratio.
