# Supplementary material for: HHLA2 in intrahepatic cholangiocarcinoma: an immune checkpoint with prognostic significance and wider expression compared with PD-L1
Source: J Immunother Cancer. 2019 Mar 18;7:77. doi: 10.1186/s40425-019-0554-8 (PMC6421676; doi:10.1186/s40425-019-0554-8)
Supplement: Supplementary file 5 — Table S5. Infiltrating patterns of immune cells with different PD-L1 expression on IC (DOCX 16 kb) [file 40425_2019_554_MOESM5_ESM.docx]

| **Table S5. Infiltrating patterns of immune cells with different PD-L1 expression on IC.** | | | |
| --- | --- | --- | --- |
| **Variables** | **PD-L1 expression** | | |
|  | **IC <1%**  **(n =127)** | **IC ≥1%**  **(n =26)** | ***P-value*** |
| CD3+TILs, (median, IQR) | 22, 9 – 40 | 47.5, 30 – 70 | **<0.001** |
| CD8+TILs, (median, IQR) | 7, 3 – 20 | 20, 5 – 31.3 | **0.006** |
| CD4+Foxp3+TILs, (median, IQR) | 3, 2 – 6 | 4, 2 – 6.25 | 0.250 |
| CD8+/CD3+ TILs ratio* (median, IQR) | 0.44, 0.20 –0.67 | 0.36, 0.21– 0.58 | 0.328 |
| CD4+Foxp3+/CD8+ TILs ratio^Ψ^ , (median, IQR) | 0.33, 0.23 – 0.63 | 0.30, 0.10 – 0.58 | 0.340 |
| CD68+ TAMs (median, IQR) | 43.5, 33.0 – 53.5 | 51.5, 41.75 – 62.63 | **0.02** |
| CD163+ TAMs (median, IQR) | 8.5, 5.0 – 15.0 | 16.0, 16.23 – 23.63 | **<0.001** |
| CD163+/CD68+ TAM ratio (median, IQR) | 0.22, 0.12 – 0.34 | 0.34, 0.21 – 0.48 | **0.001** |
| CD20+ TILs (median, IQR) | 9.0, 4.0 – 15.0 | 10.75, 5.13 – 17.63 | 0.33 |
| CD3+TIL |  |  | **0.001** |
| < 50 | 103 | 13 |  |
| ≥ 50 | 24 | 13 |  |
| CD8+TILs |  |  | 0.252 |
| < 5 | 24 | 2 |  |
| ≥ 5 | 103 | 24 |  |
| CD4+Foxp3+TILs |  |  | 0.109 |
| < 8 | 116 | 21 |  |
| ≥ 8 | 11 | 5 |  |
| CD8+/CD3+ TILs ratio* |  |  | 0.524 |
| ≤ 0.4 | 55 | 14 |  |
| > 0.4 | 63 | 12 |  |
| CD4+Foxp3+/CD8+ TILs ratio^Ψ^ |  |  | 0.612 |
| ≤ 0.6 | 73 | 19 |  |
| > 0.6 | 30 | 5 |  |

Abbreviations: IQR, interquartile range; TIL, tumor infiltrating lymphocytes; IC, immune cells. TAM, tumor associated macrophages. * The CD8+/CD3+ TILs ratio was not applicable in 9 patients with no CD3+ TILs. ^Ψ^ The CD4+Foxp3+/CD8+ TILs ratio was not applicable in 26 patients with no CD8+ TILs ratio.
