# Supplementary material for: HHLA2 in intrahepatic cholangiocarcinoma: an immune checkpoint with prognostic significance and wider expression compared with PD-L1
Source: J Immunother Cancer. 2019 Mar 18;7:77. doi: 10.1186/s40425-019-0554-8 (PMC6421676; doi:10.1186/s40425-019-0554-8)
Supplement: Supplementary file 6 — Table S6. Univariate analyses of densities of different immune cells for OS and RFS in ICC. (DOCX 17 kb) [file 40425_2019_554_MOESM6_ESM.docx]

**Table S6. Univariate analyses of densities of different immune cells for overall survival and recurrence-free survival in ICC.**

| **Variables** | **Overall survival** | |  | **Recurrence-free survival** | |
| --- | --- | --- | --- | --- | --- |
|  | **Univariate *P*-value** | **Univariate HR (95%CI)** |  | **Univariate *P*-value** | **Univariate HR (95%CI)** |
| Continuous variables |  |  |  |  |  |
| CD3+TILs | **0.013** | 0.988 (0.978 - 0.997) |  | **0.019** | 0.988 (0.979 - 0.998) |
| CD8+TILs | **<0.001** | 0.956 (0.938 - 0.975) |  | **<0.001** | 0.969 (0.953 - 0.987) |
| CD4+Foxp3+TILs | 0.075 | 1.050 (0.995 - 1.108) |  | 0.583 | 1.018 (0.956 - 1.083) |
| CD8+/CD3+ TILs ratio* | **<0.001** | 0.108 (0.047 - 0.246) |  | **<0.001** | 0.196 (0.086 - 0.448) |
| CD4+Foxp3+/CD8+ TILs ratio^Ψ^ | **<0.001** | 2.697 (1.967 - 3.698) |  | **0.009** | 1.602 (1.124 - 2.283) |
| CD68+ TAMs | 0.796 | 1.002 (0.989 - 1.014) |  | 0.888 | 1.001 (0.989 - 1.013) |
| CD163+ TAMs | 0.904 | 1.002 (0.976 - 1.027) |  | 0.392 | 0.989 (0.963 - 1.015) |
| CD163+/CD68+ TAM ratio | 0.788 | 0.856 (0.274 - 2.760) |  | 0.118 | 0.384 (0.115 -1.274) |
| CD20+ TILs | 0.461 | 1.010 (0.984 - 1.037) |  | 0.563 | 1.008 (0.981 - 1.036) |
| Dichotomized variables |  |  |  |  |  |
| CD3+TILs (≥ 50 vs < 50) | 0.067 | 0.624 (0.377 - 1.033) |  | 0.425 | 0.826 (0.517 - 1.320) |
| CD8+TILs (≥ 5 vs <5) | **0.034** | 0.591 (0.363 - 0.961) |  | **0.001** | 0.440 (0.274 - 0.705) |
| CD4+Foxp3+TILs (< 8 vs ≥ 8) | 0.055 | 1.780 (0.987 - 3.209) |  | 0.543 | 1.226 (0.636 - 2.366) |
| CD8+/CD3+ TILs ratio* (> 0.4 vs ≤ 0.4) | **<0.001** | 0.331 (0.214 - 0.513) |  | **<0.001** | 0.396 (0.258 - 0.606) |
| CD4+Foxp3+/CD8+ TILs ratio^Ψ^ (> 0.4 vs ≤ 0.4) | **<0.001** | 3.200 (1.998 - 5.125) |  | **0.005** | 2.062 (1.242 - 3.422) |
| Abbreviations: TILs, tumor infiltrating lymphocytes; TAMs, tumor associated macrophages; HR, hazard ratio; CI, confidence interval; * The CD8+/CD3+ TILs ratio was not applicable in 9 patients with no CD3+ TILs; ^Ψ^ The CD4+Foxp3+/CD8+ TILs ratio was not applicable in 26 patients with no CD8+ TILs ratio. | | | | | |
